# The Essential Oil from Cupules of *Aiouea montana* (Sw.) R. Rohde: Chemical and Enantioselective Analyses of an Important Source of (–)-α-Copaene

**DOI:** 10.3390/plants14162474

**Published:** 2025-08-09

**Authors:** Crisol F. Cueva, Yessenia E. Maldonado, Nixon Cumbicus, Gianluca Gilardoni

**Affiliations:** 1 Carrera de Bioquímica y Farmacia, Universidad Técnica Particular de Loja (UTPL), Calle Paris s/n y Praga, Loja 110107, Ecuador; cfcueva@utpl.edu.ec; 2 Programa de Doctorado en Química, Universidad Técnica Particular de Loja (UTPL), Calle Paris s/n y Praga, Loja 110107, Ecuador; yemaldonado2@utpl.edu.ec; 3 Departamento de Ciencias Biológicas y Agropecuarias, Universidad Técnica Particular de Loja (UTPL), Calle Paris s/n y Praga, Loja 110107, Ecuador; nlcumbicus@utpl.edu.ec; 4 Departamento de Química, Universidad Técnica Particular de Loja (UTPL), Calle Paris s/n y Praga, Loja 110107, Ecuador

**Keywords:** Lauraceae, volatile fraction, chiral separation, mass spectrometry, Ecuador

## Abstract

The present study described, for the first time, the chemical and enantiomeric composition of an essential oil, distilled from the cupules of *Aiouea montana* (Sw.) R. Rohde. On the one hand, chemical analyses were carried out through GC-MS (qualitative) and GC-FID (quantitative), on two stationary phases of different polarity. Major components (≥3.0%) were *S*-methyl-*O*-2-phenylethyl carbonothioate (23.1%), α-copaene (20.3%), α-phellandrene (18.7%), (*E*)-β-caryophyllene (6.1%), and α-pinene (4.5%). On the other hand, enantioselective analyses were conducted, through GC-MS, on two columns with different chiral selectors, based on derivatised β-cyclodextrins. A total of 12 chiral components were analysed, of which (1*S*,5*S*)-(−)-α-pinene and (1*R*,2*S*,6*S*,7*S*,8*S*)-(−)-α-copaene were found to be enantiomerically pure. All the other chiral components were present as scalemic mixtures. Finally, both chemical and enantiomeric profiles were compared to the ones previously described in the literature for the leaf essential oil of *A. montana*. In conclusion, cupules of *A. montana* produced an essential oil with a higher yield in comparison with leaves but with a lower content of *S*-methyl-*O*-2-phenylethyl carbonothioate. On the other hand, to some extent, the enantiomeric compositions of these volatile fractions were somewhat different. To the best of the authors’ knowledge, the cupule essential oil of *A. montana* could be the second main natural source of (−)-α-copaene so far described in the literature.

## 1. Introduction

The search for new bioactive molecules and the description of unprecedented natural products has been, for about two centuries, an important source of new pharmacologically important compounds. Nowadays, despite the rational design of active principles, nature continues to be an inspiration for medicinal chemists [1], together with the principal provider of nutraceuticals and natural aromas. In order to increase the chance of discovering new natural products, chemists are focusing on so called megadiverse countries, a group of 17 countries that have been identified by the United Nations for reuniting the greatest biodiversity around the world [2]. This group of countries includes Ecuador, where historical and logistical reasons determined that most of its native and endemic flora is still unstudied from the phytochemical point of view [3,4]. For these reasons, our group has been studying Ecuadorian biodiversity for many years, recently focusing on the description of unprecedented essential oils [5,6,7,8], mainly concerning their chemical compositions, enantiomeric profiles, biological activities, and olfactometric properties.

The present research focused on the chemical and enantioselective analysis of an essential oil (EO), distilled from the dry cupules of *Aiouea montana* (Sw.) R. Rohde (Lauraceae). This plant, a quite diffuse neotropical species, has been the object of a recent publication, where a high-yield EO from leaves has been described for the first time [9]. On that occasion, the authors were attracted by the strong, sulphurous, unpleasant odour of this plant, for which no literature was reported. The smell was attributed to the presence of *S*-methyl-*O*-2-phenylethyl carbonothioate, the main component of the EO, that was described in nature at that moment for the first time [9]. Due to the shape of its fruits, this plant is popularly known in Ecuador as “aguacatillo”. However, to the best of the authors’ knowledge and despite the high diffusion of this species, no ethnobotanical or gastronomic traditional use is reported for *A. montana.*

**Figure 1:**
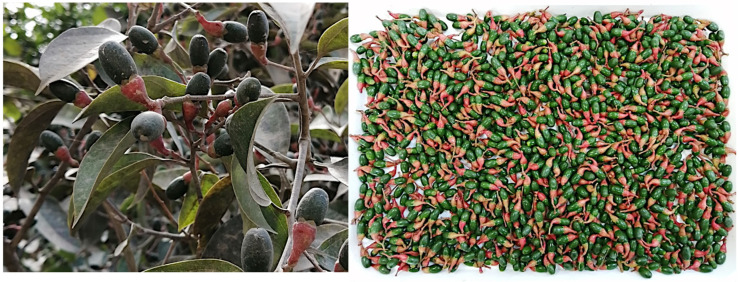
Leaves and fruits of *A. montana* at the collection site (**left**) and before drying (**right**). Cupules are the conical, red structures that can be observed between fruits and stems (photo: Gianluca Gilardoni).

After analysing the leaves, the authors of the present study observed that cupules, the red structures connecting fruits and stems (see Figure 1), also presented an intense sulphurous odour, very similar but apparently not identical to that of the leaves. This phenomenon is not unprecedented within the family Lauraceae, and it is for instance well known in *Ocotea quixos* (Lam.) Kosterm, a cinnamon-like Amazonian species, commonly known with the traditional name “ishpingo”. The leaves, bark, and cupules of *O. quixos* all produce an EO, similar in odour but different in composition [10]. The difference is so relevant that, despite leaves being more economic, cupules are the real “ishpingo” spice in Ecuadorian gastronomy. The aim of the present study was to investigate if a similar difference existed between dry leaves and dry cupules of *A. montana*. Some notions about the botany of this species have already been reported in the previous study [9]. Concerning cupules, they are a typical morphological structure within the family Lauraceae. In this family, flowers typically exhibit a perigynous structure, that characterize the hypanthium. Hypanthium often enlarges as the fruit develops, forming structures that partially enclose or support the mature fruits [11]. These structures are cupules. In the present study, a special emphasis has been kept on the enantiomeric composition of this volatile fraction, as a critical aspect that should be considered in all EO analysis. It is in fact well known that the two enantiomeric forms of a same molecule, despite presenting the same physical and chemical properties (except for the optical rotatory power), are often characterised by different biological activities. This phenomenon is explained by the interaction of enantiomers with chiral primary metabolites, such as enzymes and membrane receptors, that actually are proteins. Two enantiomers do not show the same affinity for a same chiral substrate, producing a different effect because of their interaction. It is therefore possible that two enantiomers present different pharmacological or physiological properties, such as different toxicity or a different aroma. For this reason, an exhaustive EO description should always include the enantioselective analysis of at least some important chiral components, in order to explain or predict biological effects that are not explained by a simple chemical analysis. Another important result of enantioselective analyses is the possible identification of enantiomerically pure major compounds. In this case, if the distillation yield is high enough, the EO could become a good source for the preparative isolation of pure enantiomers. Enantiomerically pure compounds are economically important as analytical standards or as chiral building blocks in fine chemical synthesis [12].

## 2. Results

### 2.1. Chemical Composition of the EO

**Figure 2:**
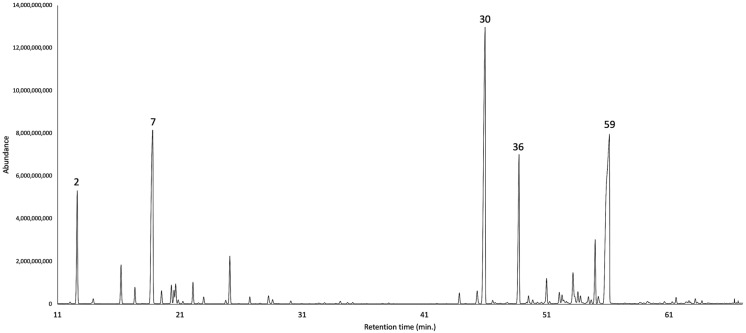
GC-MS profile of *A. montana* cupule EO on a 5%-phenyl-methylpolysiloxane stationary phase. The peak numbers refer to major compounds (≥3.0% as average amount) in Table 1: α-pinene (**2**), α-phellandrene (**7**), α-copaene (**30**), (*E*)-β-caryophyllene (**36**), and *S*-methyl-*O*-2-phenylethyl carbonothioate (**59**).

**Figure 3:**
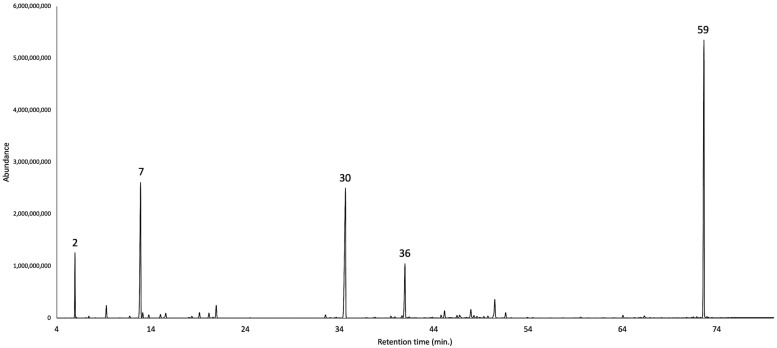
GC-MS profile of *A. montana* cupule EO on a polyethylene glycol stationary phase. The peak numbers refer to major compounds (≥3.0% as average amount) in Table 1: α-pinene (**2**), α-phellandrene (**7**), α-copaene (**30**), (*E*)-β-caryophyllene (**36**), and *S*-methyl-*O*-2-phenylethyl carbonothioate (**59**).

**Figure 4:**
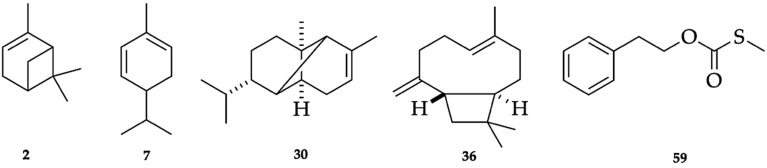
Major components (≥3.0% as average amount) of *A. montana* cupule EO. The numbers refer to Table 1: α-pinene (**2**), α-phellandrene (**7**), α-copaene (**30**), (*E*)-β-caryophyllene (**36**), and *S*-methyl-*O*-2-phenylethyl carbonothioate (**59**).

The dry cupules of *A. montana*, analytically distilled, produced an EO with a yield of 2.7 ± 0.45% by weight, significantly higher than the one of dry leaves (1.6%) [9]. This datum is consistent with results previously obtained from *O. quixos*, within the same family Lauraceae, where cupules also were the most high-yielding structures (1.8% versus 1.0% and 1.5% for bark and leaves, respectively) [10]. A total of 81 metabolites were detected and quantified on at least one of two columns, with stationary phases of different polarity. As an average value on the two columns, all these components corresponded to 97.4% of the whole oil mass. Major constituents (≥3.0% as mean value) were *S*-methyl-*O*-2-phenylethyl carbonothioate (23.1%, **59**), α-copaene (20.3%, **30**), α-phellandrene (18.7%, **7**), (*E*)-β-caryophyllene (6.1%, **36**), and α-pinene (4.5%, **2**). Monoterpenes and sesquiterpenes almost equally contributed to the EO composition, accounting for 32.1% and 38.0%, respectively, of the total EO mass. Figure 2 and Figure 3 represent the GC profiles of *A. montana* cupule EO, on one non-polar and one polar stationary phase, respectively. The major compounds are represented in Figure 4, whereas the complete qualitative and quantitative analyses are detailed in Table 1.

### 2.2. Enantioselective Analysis

The enantioselective analysis was carried out through two different columns, whose stationary phases were based on derivatised β-cyclodextrins. The choice of the chiral selectors depended on the analytes, since different enantiomeric pairs properly separated on different stationary phases. Furthermore, due to the partial superposition of the chiral compounds with other constituents, some integrations were achieved by extracting specific ions instead of using the total ion current, as reported in Table 2. A total of 12 chiral components were analysed, of which (1*S*,5*S*)-(−)-α-pinene and (1*R*,2*S*,6*S*,7*S*,8*S*)-(−)-α-copaene were found to be enantiomerically pure. All the other chiral components were present as scalemic mixtures, although (1*R*,5*R*)-(+)-β-pinene, (*R*)-(−)-linalool, (1*R*,2*S*,4*R*)-(+)-borneol, and (*S*)-(−)-α-terpineol showed an enantiomeric excess (e.e.) higher than 80%. On the other hand, (*S*)-(+)-terpinen-4-ol manifested the lowest e.e. (8.6%), approaching to racemate.

## 3. Discussion

**Figure 5:**
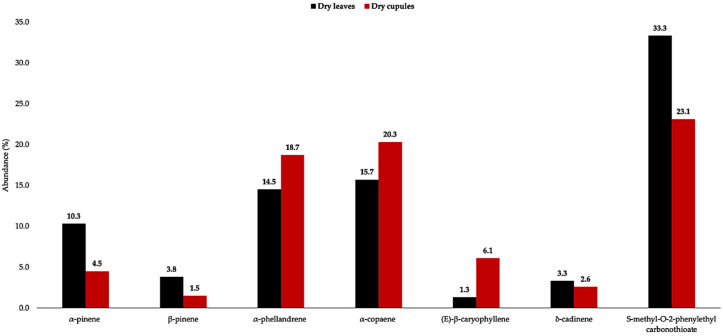
Compared amounts of major compounds (≥3.0% in at least one oil) in dry leaf (black) and dry cupule (red) EOs of *A. montana*.

If the major constituents of dry leaves and dry cupules are compared (see Figure 5), a close relationship between the two profiles is observed [9]. The main difference was the lower amount of *S*-methyl-*O*-2-phenylethyl carbonothioate (**59**) that corresponded to a higher contribution of α-copaene (**30**), α-phellandrene (**7**), and (*E*)-β-caryophyllene (**36**). On the one hand, compound **59** was the principal responsible for the sulphurous odour, and its lower percentage could justify a lower smell intensity. On the other hand, α-copaene (**30**) was almost as abundant as *S*-methyl-*O*-2-phenylethyl carbonothioate (**59**), resulting in a possible greater contribution to the whole aromatic profile. Another difference is represented by the amounts of pinenes, which are significantly lower in dry cupules than in dry leaves.

**Figure 6:**
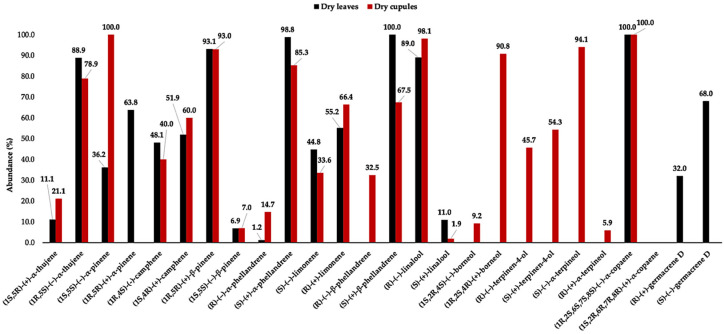
Compared enantiomeric profiles of some chiral compounds in dry leaf (black) and dry cupule (red) EOs of *A. montana*.

In contrast to chemical profiles, the enantiomeric compositions of dry leaf and dry cupule EOs were somewhat different, despite similarities being observed (see Figure 6) [9]. The difference did not lie only in the presence of chiral compounds that were specific to each oil, such as borneol, terpinen-4-ol, and terpineol for cupules and germacrene D for leaves, but also in the significantly different e.e. for some common components. This was mainly the case of α-pinene and β-phellandrene. Regarding α-pinene, leaves were dominated by the dextrorotatory form, whereas cupules presented the laevorotatory isomer as a pure enantiomer. Concerning β-phellandrene, the dextrorotatory enantiomer was enantiomerically pure in leaves, but it showed an e.e. of only 35.0% in cupules. To a lesser extent, α-phellandrene also presented some difference, with (*S*)-(+)-α-phellandrene showing an e.e of 97.6% in dry leaves and 70.6% in dry cupules. These discrepancies affirm that, regarding volatile fractions, different morphological structures can produce different enantiomeric profiles, even when the chemical compositions are similar. Enantioselectivity in biosynthetic pathways is a well-known phenomenon, which can be explained by the involvement of enzymes as chiral catalysts and justified by the different biological properties that are exerted by different enantiomers [68]. Also, different metabolic profiles, observed in different organs, are a typical trend in secondary metabolism. This phenomenon can be explained by the different needs and functions of organs and morphological structures in general. For example, it has been demonstrated that the chemical profile of an EO can be related to the bacterial phytobiome, associated with different compartments of a plant, with ecological implications [69]. Finally, an enzymatic effect on the enantiomeric composition cannot be excluded during drying, as suggested in the previous study on this species [9].

Concerning the biological activities of the main components, no information exists, to best of the authors’ knowledge, about *S*-methyl-*O*-2-phenylethyl carbonothioate (**59**). However, the biological properties of the other abundant metabolites have been described in the literature. Even if they are not always pure compounds, their biological properties have been studied at least as mixtures in EOs where they were found to be abundant. For instance, α-copaene (20.3%, **30**) has been detected as a major compound in some EOs, such as *Dipteryx alata* fruit EO (21.8%, main component), *Polyalthia suberosa* leaf EO (15.5%, main component), *Cinnamomum cassia* bark EO (15.7%, second most abundant constituent), the dry leaf EO of *A. montana* itself (15.7%, second most abundant constituent), *Araucaria heterophylla* oleoresin EO (10.8%, main component), and *Trixis michuacana* var. *longifolia* aerial parts EO (9.91%, second most abundant constituent), among others [9,70,71,72,73,74]. According to some of these sources, the following biological activities could be attributed to these volatile fractions and, indirectly, hypothesised for α-copaene: antibacterial (especially against *Staphylococcus* spp.), cholinergic, anti-inflammatory, phytotoxic, and antioxidant [71,72,73,74]. Furthermore, α-copaene is surely an enantiospecific attractive for pest insect *Ceratitis capitata*, whereas it acts as a repellent for other insect species, such as fire ants (*Solenopsis invicta*) [75,76,77].

The following most abundant component of cupule EO is α-phellandrene (18.7%, **7**), one of the most prevalent monoterpenes found in EOs. Among the reported activities, the most notable one is arguably the in vivo antinociceptive effect, observed in rodent models and corroborated by a study demonstrating its antihyperalgesic activity [78,79]. While α-phellandrene appears to lack significant in vitro antimicrobial activity, it has been shown to enhance macrophage phagocytosis and the cytotoxic activity of natural killer cells [80]. Moreover, α-phellandrene has been reported to induce DNA damage in murine leukaemia cells, concurrently impairing their DNA repair mechanisms in in vitro conditions [81,82].

After α-phellandrene (**7**), (*E*)-β-caryophyllene (**36**) is the most abundant compound (20.3%) in *A. montana* cupule EO. Mechanistically, **36** functions as a selective agonist of the cannabinoid receptor type 2 (CB2), also exhibiting modulatory interactions with members of the peroxisome proliferator-activated receptor (PPAR) family, notably PPAR-α and PPAR-γ. Through these molecular engagements, (*E*)-β-caryophyllene (**36**) has been shown to elicit pronounced anti-inflammatory effects, principally via the downregulation of key pro-inflammatory cytokines and transcription factors. In addition to its immunomodulatory potential, compound **36** has demonstrated significant neuroprotective efficacy in preclinical models of Alzheimer’s disease [83,84].

Finally, the last major component is α-pinene (4.5%, **2**). Compound **2**, a highly abundant monoterpene, has been extensively documented for its multifaceted biological activities. Among these, its potent antibacterial efficacy is particularly noteworthy, with demonstrable activity against multidrug-resistant bacterial strains, including methicillin-resistant *Staphylococcus aureus* (MRSA). Furthermore, α-pinene possesses significant antifungal properties, especially against *Candida* spp. In the context of inflammation, this monoterpene has been shown to attenuate the expression of key pro-inflammatory mediators, thereby exhibiting anti-inflammatory potential. Neuropharmacological investigations have additionally identified α-pinene (**2**) as a neuroprotective agent, capable of ameliorating cognitive impairment in scopolamine-induced models of memory dysfunction. Beyond its neuroprotective profile, α-pinene (**2**) has also demonstrated anticonvulsant and anti-leishmanial properties in relevant experimental systems [85].

Considering that the biological activities of chiral compounds may depend on stereochemistry, the previous information should possibly correlate to the results of the enantioselective analysis. In the present EO, (1*S*,5*S*)-(−)-α-pinene and (1*R*,2*S*,6*S*,7*S*,8*S*)-(−)-α-copaene were enantiomerically pure, and (*S*)-(+)-α-phellandrene presented a high e.e., whereas no chiral information could be associated with (*E*)-β-caryophyllene (**36**). Regarding α-copaene (**30**), it is certainly an enantioselective semiochemical, whose dextrorotatory isomer is the most attractive form for *C. capitata*, whereas the laevorotatory enantiomer is specifically repellent for *S. invicta* [75,76,77]. Concerning (*S*)-(+)-α-phellandrene, no information has been found in the literature about any enantioselective biological activity. On the other hand, α-pinene (**2**) has been investigated for its enantioselective biological properties. According to the literature, whereas the dextrorotatory isomer demonstrated a wider range of antimicrobial and anti-inflammatory activities, (1*S*,5*S*)-(−)-α-pinene is notable for its antiviral capacity and its ability to modulate antibiotic resistance. Both enantiomers shared a potential neuroprotective effect through the inhibition of acetylcholinesterase [84].

## 4. Materials and Methods

### 4.1. Plant Material

The fruits of *A. montana* were collected on 9 November 2023, from the same trees that provided leaves for the study previously published by these authors [9]. The plant was distributed in a range of about 300 m around a central point, with coordinates 3°49′56″ S and 79°28′41″ W, at an altitude of about 1780 m above the sea level. The whole fruits were washed (see Figure 1) and dried at 35 °C for 48 h. After that, dry cupules were separated from fruits, obtaining 58.0 g of dry plant material. In order to ensure that leaf EO data could correctly be compared with the ones from cupules, a small number of leaves was also collected, dried, and distilled, producing a volatile fraction with a GC profile identical to the one previously described in the literature. The taxonomical identification was performed by one of the authors (N.C.), based on morphological criteria and comparison with herbarium specimens. A botanical voucher corresponding to this species is deposited at the herbarium of the Universidad Técnica Particular de Loja, with code 14997. Both collection and investigation were conducted by appointment of the Ministry of Environment, Water, and Ecological Transition of Ecuador (MAATE), with permit code MAATE-DBI-CM-2022-0248.

### 4.2. Distillation and Sample Preparation

The entire number of dry cupules was divided into four equal portions (14.5 g each) and analytically steam-distilled in four repetitions, in a modified Dean–Stark apparatus, as previously described in the literature [86]. In this process, 2 mL of cyclohexane were located upon the water phase inside the distillation apparatus, in order to extract the organic volatile fraction. The cyclohexane was spiked with *n*-nonane as an internal standard, at the concentration of 0.7 mg/mL. Both cyclohexane and internal standard were purchased from Merk (Sigma-Aldrich, St. Louis, MO, USA). After 4 h, the four cyclohexane solutions were recovered and permanently stored at −15 °C, to be directly submitted to gas chromatography.

### 4.3. Qualitative (GC-MS) Chemical Analyses

Qualitative analyses were conducted on a Trace 1310 gas chromatograph (GC), coupled with an ISO 7000 single quadrupole mass spectrometer (MS). The whole GC-MS system was provided by Thermo Fisher Scientific (Walthan, MA, USA). All the analyses were repeated on two capillary columns, coated with 5% phenyl methyl polysiloxane (TR-5MS, non-polar) and polyethylene glycol (TR-Wax, polar), both purchased from Thermo Fisher Scientific (Walthan, MA, USA). These columns were 30 m long, with an internal diameter of 0.25 mm and a phase thickness of 0.25 μm. The columns reached the detector through a transfer line set at 250 °C, whereas the injector was maintained at 230 °C and operated in split mode, with a split ratio of 40:1. The carrier gas was helium, maintained at the constant flow of 1 mL/min, and provided by Indura S.A. (Guayaquil, Ecuador). The elutions were conducted according to the following thermal program: 50 °C for 10 min, followed by a first gradient of 2 °C/min until 155 °C, and a second gradient of 5 °C/min until 230 °C, which was maintained for 5 min. The injection volume was 1 μL. The ion source was an electron impact device, set at 70 eV, whose temperature was programmed at 250 °C, as well as the quadrupolar mass analyser. The MS was operated in SCAN mode, in the range 40–400 *m/z*. With both columns, all compounds were identified by comparison of each mass spectrum and linear retention index (LRI) with data from the literature (see Table 1). The LRIs were calculated according to Van den Dool and Kratz [87], based on a mixture on *n*-alkanes in the range C_9_–C_24_.

### 4.4. Quantitative (GC-FID) Chemical Analyses

Quantitative analyses were performed with the same GC instrument used for qualitative profiling, equipped with the same two columns and operated with the same temperatures, carrier gas flow, thermal program, and injection volume. On the other hand, a flame ionization detector (FID) was used instead of MS, whereas the split ratio was 10:1. All the identified compounds were quantified using two six-point calibration curves, one for each column, with isopropyl caproate as calibration standard and *n*-nonane as internal standard. The calibration standard was synthetised in the authors’ laboratory and purified until 98.8% (GC-FID purity), whereas *n*-nonane was purchased from Merck (Sigma-Aldrich, St. Louis, MO, USA). The six dilutions were prepared as previously described in the literature [88], obtaining curves with *R*^2^ > 0.998. All the quantitative results were expressed as the mean values and standard deviations of four repetitions (see Section 4.2). Before applying to calibration curves, each integration area was transformed by using a relative response factor (RRF), calculated on the basis of the combustion enthalpy, as described in the literature [89,90].

### 4.5. Enantioselective Analyses

Enantioselective analyses were performed employing two capillary columns, both featuring stationary phases based on 2,3-diacetyl-6-*tert*-butyldimethylsilyl-β-cyclodextrin and 2,3-diethyl-6-*tert*-butyldimethylsilyl-β-cyclodextrin as chiral selectors. Each column measured 25 m in length, with an internal diameter of 0.25 mm and a film thickness of 0.25 µm (Mega s.r.l., Milan, Italy).

The analyses were carried out using the same GC–MS system previously described for qualitative profiling, operating under the following thermal gradient: initial oven temperature of 50 °C held for 1 min, ramped at 2 °C/min to 220 °C, and held isothermal for 10 min. Helium was used as the carrier gas at constant pressure (70 kPa). All instrumental settings mirrored those applied in the qualitative analyses, except for the injector and transfer line temperatures, both maintained at 220 °C.

Enantiomeric identification was achieved through a combination of MS data and linear retention indices (LRIs), the latter calculated according to the Van den Dool and Kratz [87]. Analytical results were compared with those obtained from injections of enantiomerically pure standards. These standards were acquired either from Merck (Sigma–Aldrich, St. Louis, MO, USA) or obtained from internal repositories at the University of Turin, Italy.

## 5. Conclusions

The dry cupules of *A. montana* produced an EO, with a distillation yield much higher than the one of leaves, confirming the importance of cupules as a source of EOs within the family Lauraceae. The chemical composition is similar to the one of leaf volatile fraction, except for the amount of *S*-methyl-*O*-2-phenylethyl carbonothioate (**59**). For this reason, despite the lowest distillation yield, fresh leaves are confirmed to be a better source of this sulphurated metabolite. On the other hand, cupules are a better source of α-copaene (**30**), and to the best of the authors’ knowledge, this EO could be the second main natural source of (–)-α-copaene so far described in the literature. This aspect is quite important, considering the scarce commercial availability of enantiomerically pure standards and the high economical cost of this kind of compounds. Furthermore, enantiomerically pure secondary metabolites can constitute important chiral building blocks in fine chemical synthesis. As for *A. montana* leaf EO, further studies should experimentally investigate the biological activities of this EO and its pure components, with a special emphasis on (−)-α-copaene and on the completely unstudied *S*-methyl-*O*-2-phenylethyl carbonothioate (**59**).

## Figures and Tables

**Table 1:** Qualitative (GC-MS) and quantitative (GC-FID) chemical composition of *A. montana* cupule EO on 5%-phenyl-methylpolysiloxane and polyethylene glycol stationary phases. Major components (≥3.0% as average value) are reported in bold.

N.	Compounds	5% Phenyl Methyl Polysiloxane					Polyethylene Glycol					Average %
		Calc.	Ref.	%	σ	Lit.	Calc.	Ref.	%	σ	Lit.	
1	α-thujene	926	924	trace	-	[13]	1022	1022	0.1	0.01	[14]	**0.1**
2	**α-pinene**	933	932	4.8	1.05	[13]	1018	1018	4.2	0.69	[15]	**4.5**
3	α-fenchene	948	945	trace	-	[13]	1050	1050	trace	-	[16]	**trace**
4	camphene	950	946	0.6	0.53	[13]	1058	1057	0.7	0.42	[17]	**0.7**
5	β-pinene	978	974	1.6	0.55	[13]	1106	1106	1.3	0.50	[18]	**1.5**
6	myrcene	992	988	0.6	0.40	[13]	1166	1166	17.9	1.49	[19]	**0.6**
7	**α-phellandrene**	1010	1002	18.7	1.60	[13]	1163	1162			[20]	**18.7**
8	α-terpinene	1019	1014	0.5	0.06	[13]	1176	1176	0.4	0.05	[21]	**0.4**
9	*o*-cymene	1029	1022	0.7	0.08	[13]	1269	1268	0.6	0.18	[22]	**0.7**
10	limonene	1032	1024	0.6	0.05	[13]	1196	1196	0.5	0.04	[20]	**0.6**
11	β-phellandrene	1033	1025	0.9	0.08	[13]	1205	1205	0.8	0.07	[23]	**0.9**
12	1,8-cineole	1036	1036			[24]	1202	1200			[25]	
13	(*Z*)-β-ocimene	1041	1032	0.1	0.00	[13]	1243	1243	0.1	0.01	[26]	**0.1**
14	(*E*)-β-ocimene	1051	1044	0.9	0.07	[13]	1255	1255	0.7	0.13	[23]	**0.8**
15	γ-terpinene	1061	1054	0.3	0.02	[13]	1243	1243	0.2	0.02	[27]	**0.3**
16	terpinolene	1084	1086	0.2	0.02	[13]	1275	1276	0.1	0.01	[28]	**0.2**
17	*p*-mentha-2,4(8)-diene	1088	1085	2.0	0.35	[13]	1280	1286	1.8	0.30	[29]	**1.9**
18	linalool	1109	1109	0.3	0.02	[30]	1562	1562	0.3	0.01	[31]	**0.3**
19	phenyl ethyl alcohol	1128	1127	0.3	0.19	[32]	1923	1923	0.3	0.19	[33]	**0.3**
20	*cis*-*p*-menth-2-en-1-ol	1133	1129	0.1	0.10	[34]	1566	-	0.2	0.03	§	**0.2**
21	*trans*-*p*-menth-2-en-1-ol	1152	1148	0.1	0.02	[35]	1635	-	0.2	0.03	§	**0.2**
22	camphene hydrate	1164	1157	trace	-	[36]	1600	-	trace	-	§	**trace**
23	borneol	1182	1179	0.1	0.01	[37]	1536	-	0.1	0.10	§	**0.1**
24	terpinen-4-ol	1189	1189	trace	-	[38]	1607	1607	trace	-	[39]	**trace**
25	*p*-cymen-9-ol	1201	1204	trace	-	[13]	1863	-	0.1	0.01	§	**0.1**
26	α-terpineol	1206	1186	0.2	0.01	[13]	1702	1700	0.2	0.05	[40]	**0.2**
27	*trans*-piperitol	1220	1207	0.2	0.02	[41]	1682	1679	0.7	0.10	[42]	**0.5**
28	carvotanacetone	1261	1256	0.1	0.01	[43]	1672	1669	0.3	0.02	[44]	**0.2**
29	α-cubebene	1345	1348	0.4	0.03	[13]	1450	1450	0.5	0.01	[45]	**0.5**
30	**α-copaene**	1377	1373	19.8	1.28	[13]	1483	1482	20.7	1.86	[46]	**20.3**
31	2-*epi*-α-funebrene	1387	1380	0.1	0.05	[13]	1535	-	0.2	0.02	§	**0.2**
32	β-elemene	1390	1389	trace	-	[13]	2004	-	trace	-	§	**trace**
33	β-isocomene	1396	1407	0.4	0.69	[13]	1457	-	0.3	0.18	§	**0.4**
34	sibirene	1405	1400	0.1	0.01	[13]	1621	-	0.1	0.01	§	**0.1**
35	longifolene	1410	1407	trace	-	[13]	1623	1623	trace	-	[47]	**trace**
36	**(*E*)-β-caryophyllene**	1421	1417	6.5	0.51	[13]	1587	1587	5.7	3.21	[48]	**6.1**
37	β-copaene	1430	1430	1.0	0.75	[13]	1651	-	0.5	0.05	§	**0.5**
38	α-*trans*-bergamotene	1433	1432			[13]	1582	1582	0.7	0.19	[49]	**0.7**
39	aromadendrene	1439	1439	0.2	0.02	[13]	1595	-	0.2	0.07	§	**0.2**
40	(*Z*)-β-farnesene	1442	1440	trace	-	[13]	1566	-	0.2	0.02	§	**0.2**
41	2-phenyl ethyl butanoate	1445	1439	0.1	0.01	[13]	2014	-	trace	-	§	**0.1**
42	*trans*-muurola-3,5-diene	1450	1451	trace	-	[13]	1602	-	0.1	0.01	§	**0.1**
43	α-humulene	1457	1452	1.0	0.08	[13]	1657	-	1.1	0.24	§	**1.1**
44	9-*epi*-(*E*)-caryophyllene	1461	1464	0.1	0.01	[13]	1604	-	trace	-	§	**0.1**
45	*trans*-cadina-1(6),4-diene	1473	1475	0.5	0.05	[13]	1651	-	0.4	0.04	§	**0.5**
46	γ-muurolene	1477	1478	0.5	0.04	[13]	1680	1680	0.5	0.04	[50]	**0.5**
47	γ-curcumene	1479	1481	0.1	0.00	[13]	1687	1685	0.1	0.08	[51]	**0.1**
48	*trans*-muurola-4(14),5-diene	1483	1493	0.1	0.01	[52]	1697	1706	0.2	0.04	[46]	**0.2**
49	β-selinene	1491	1489	1.6	0.34	[13]	1706	1706	1.5	0.56	[53]	**1.6**
50	α-zingiberene	1494	1493			[13]	1659	-			§	
51	α-selinene	1498	1498	0.9	0.08	[13]	1711	1741	0.5	0.35	[53]	**0.7**
52	α-muurolene	1499	1500			[13]	1717	-			§	
53	δ-amorphene	1503	1511	0.1	0.02	[13]	1752	-	0.9	1.70	§	**0.5**
54	isodaucene	1508	1500	0.2	0.13	[13]	1755	-	trace	-	§	**trace**
55	β-curcumene	1510	1514			[13]	1739	1743	0.3	0.02	[54]	**0.3**
56	γ-cadinene	1514	1513	0.1	0.01	[13]	1700	-	trace	-	§	**0.1**
57	δ-cadinene	1520	1522	2.2	0.31	[13]	1752	1752	3.0	0.51	[23]	**2.6**
58	zonarene	1524	1528	0.1	0.15	[13]	1749	-	trace	-	§	**0.1**
59	***S*** **-methyl-*O*-2-phenylethyl carbonothioate**	1539	1538	23.1	5.81	[9]	2223	-	22.5	4.44	§	**23.1**
60	α-cadinene	1543	1537	0.1	0.01	[13]	2227	-	0.1	0.01	§	**0.1**
61	germacrene B	1561	1559	trace	-	[13]	1713	-	0.2	0.07	§	**0.2**
62	caryolan-8-ol	1581	1571	0.1	0.01	[13]	2047	-	0.2	0.02	§	**0.2**
63	spathulenol	1583	1577			[13]	2127	2128			[28]	
64	caryophyllene oxide	1587	1582	trace	-	[13]	1970	1970	trace	-	[55]	**trace**
65	gleenol	1592	1586	0.2	0.01	[13]	2038	2035	0.3	0.04	[56]	**0.3**
66	2-phenyl ethyl tiglate	1595	1590			[57]	2196	2190			[58]	
67	guaiol	1606	1600	trace	-	[13]	2065	2064	0.1	0.01	[59]	**0.1**
68	γ-eudesmol	1638	1630	0.1	0.01	[60]	2097	-	0.1	0.01	§	**0.1**
69	β-eudesmol	1641	1649	trace	-	[13]	2104	-	trace	-	§	**trace**
70	1-*epi*-cubenol	1645	1638	0.3	0.07	[61]	2059	2060	0.4	0.02	[51]	**0.4**
71	allo-aromadendrene epoxide	1654	1645	trace	-	[62]	2152	-	trace	-	§	**trace**
72	cubenol	1662	1651	trace	-	[63]	2052	2052	0.2	0.02	[46]	**0.2**
73	α-muurolol (=torreyol)	1664	1668	0.1	0.01	[64]	2178	2178	0.4	0.07	[65]	**0.3**
74	α-cadinol	1667	1666			[42]	2191	2191			[54]	
75	7-*epi*-α-eudesmol	1670	1662	0.1	0.01	[13]	2207	2205	trace	-	[62]	**0.1**
76	intermedeol	1682	1674	0.2	0.01	[43]	2261	2264	0.1	0.01	[66]	**0.2**
77	*epi*-β-bisabolol	1690	1670	trace	-	[13]	2163	-	0.1	0.01	§	**0.1**
78	α-bisabolol	1700	1699	0.1	0.01	[67]	2076	-	trace	-	§	**0.1**
79	*epi*-α-bisabolol	1701	1683	trace	-	[13]	2324	-	trace	-	§	**trace**
80	eudesm-7(11)-en-4-ol	1713	1709	trace	-	[44]	2258	-	trace	-	§	**trace**
81	(2*Z*,6*E*)-farnesol	1731	1722	trace	-	[13]	2252	-	trace	-	§	**trace**
	monoterpenes			32.5					29.4			**32.0**
	oxygenated monoterpenoids			1.1					2.1			**1.8**
	sesquiterpenes			36.1					38.0			**38.0**
	oxygenated sesquiterpenoids			1.2					1.9			**2.1**
	others			23.5					22.8			**23.5**
	total			94.4					94.2			**97.4**

N. = progressive number; Calc. = calculated linear retention index (see Section 4.3); Ref. = reference linear retention index according to the literature (Lit.); Lit. = reference literature for linear retention indices; % = percent by weight of EO; σ = standard deviation; § = identification by MS only; trace = < 0.1%; Average % = mean amount between the two columns. If in one column the component is trace, undetected, or sum of two peaks, only the value of the other column is reported.

**Table 2:** Enantioselective analysis of some chiral terpenes from *A. montana* cupule EO.

Chiral Selector	Ion Integration	Enantiomer	LRI	E.D. (%)	e.e. (%)
DET	TIC	(1*S*,5*R*)-(+)-α-thujene	915	21.1	57.8
DET	TIC	(1*R*,5*S*)-(−)-α-thujene	919	78.9	
DAC	TIC	(1*S*,5*S*)-(−)-α-pinene	914	100.0	100.0
DAC	TIC	(1*R*,5*R*)-(+)-α-pinene	916	-	
DET	TIC	(1*R*,4*S*)-(−)-camphene	922	40.0	20.0
DET	TIC	(1*S*,4*R*)-(+)-camphene	938	60.0	
DET	TIC	(1*R*,5*R*)-(+)-β-pinene	950	93.0	86.0
DET	TIC	(1*S*,5*S*)-(−)-β-pinene	961	7.0	
DET	TIC	(*R*)-(−)-α-phellandrene	1018	14.7	70.6
DET	TIC	(*S*)-(+)-α-phellandrene	1021	85.3	
DET	68 (*m/z*)	(*S*)-(−)-limonene	1060	33.6	32.8
DET	68 (*m/z*)	(*R*)-(+)-limonene	1076	66.4	
DET	TIC	(*R*)-(−)-β-phellandrene	1053	32.5	35.0
DET	TIC	(*S*)-(+)-β-phellandrene	1064	67.5	
DET	71 (*m/z*)	(*R*)-(−)-linalool	1182	98.1	96.2
DET	71 (*m/z*)	(*S*)-(+)-linalool	1196	1.9	
DET	95 (*m/z*)	(1*S*,2*R*,4*S*)-(−)-borneol	1205	9.2	81.6
DET	95 (*m/z*)	(1*R*,2*S*,4*R*)-(+)-borneol	1213	90.8	
DAC	71 (*m/z*)	(*R*)-(−)-terpinen-4-ol	1291	45.7	8.6
DAC	71 (*m/z*)	(*S*)-(+)-terpinen-4-ol	1297	54.3	
DET	59 (*m/z*)	(*S*)-(−)-α-terpineol	1302	94.1	88.2
DET	59 (*m/z*)	(*R*)-(+)-α-terpineol	1314	5.9	
DET	TIC	(1*R*,2*S*,6*S*,7*S*,8*S*)-(−)-α-copaene	1322	100.0	100.0
DET	TIC	(1*S*,2*R*,6*R*,7*R*,8*R*)-(+)-α-copaene	1324	-	

DET = 2,3-diethyl-6-*tert*-butyldimethylsilyl-β-cyclodextrin; DAC = 2,3-diacetyl-6-*tert*-butyldimethylsilyl-β-cyclodextrin; TIC = total ion current; LRI = calculated linear retention index; E.D. = enantiomer distribution; e.e. = enantiomeric excess.

## Data Availability

The datasets presented in this article are not readily available, because they are part of an ongoing study. Requests to access the datasets should be directed to the corresponding author.
